# Development of Cell-Permeable Adenylosuccinate Lyase Inhibitor

**DOI:** 10.3390/mps8060126

**Published:** 2025-10-29

**Authors:** Yijia Hu, Young-Sam Lee

**Affiliations:** 1 Department of Biology, Johns Hopkins University, Baltimore, MD 21218, USA; 2 Department of Family Medicine, Boston University Chobanian and Avedisian School of Medicine, Boston, MA 02118, USA; 3 Department of Molecular and Cellular Biochemistry, University of Kentucky, Lexington, KY 40536, USA

**Keywords:** adenylosuccinate lyase, inhibitor, screening, nucleotide

## Abstract

Abnormal adenylosuccinate lyase (ADSL) activity is associated with cancer and neurodevelopmental processes. However, a cell-permeable ADSL inhibitor is not yet available. Our high-throughput screen identified NF-449 as a potential lead compound. To improve cell permeability of the lead compound, fragments of NF-449 were synthesized. This fragment, 2,2’-(1,3-phenylenebis(carbonylimino))-bisbenzenesulfonate, competitively inhibits purified human ADSL with an inhibitory constant of 0.4 micromolar. Its triethylammonium salt inhibited ADSL in HeLa cells with an IC_50_ of 0.4 micromolar. While this compound might not be ready for in vivo applications yet, further improvement in its permeability might produce useful reagents for in vivo studies of ADSL.

## 1. Introduction

**Figure 1:**
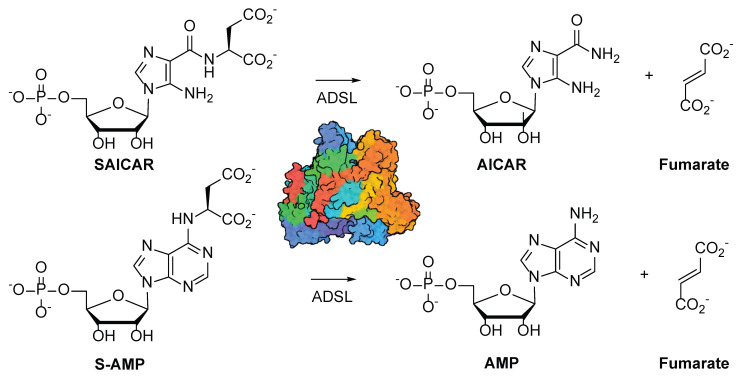
Reactions catalyzed by ADSL. ADSL catalyzes two reactions, resulting in the cleavage of SAICAR and S-AMP to AICAR and AMP, respectively. Fumarate is produced in both reactions.

Adenylosuccinate lyase (ADSL, EC 4.3.2.2) is a conserved enzyme catalyzing two reactions in the de novo purine nucleotide biosynthesis pathway (Figure 1) [1,2,3]. ADSL catalyzes the cleavage of *N*-succinyl-5-aminoimidazole-4-carboxamide-1-ribose-5’-monophosphate (SAICAR) to 5-aminoimidazole-4-carboxamide-1-ribose-5’-monophosphate (AICAR) and fumarate. Downstream in the pathway, ADSL also catalyzes the cleavage of adenylosuccinate (S-AMP), producing adenosine 5’-monophosphate (AMP) along with fumarate.

Abnormal ADSL activity has been associated with human diseases. The best-known example is ADSL deficiency (OMIM 608222) [2,3]. This disease is a rare recessive inborn error in metabolism caused by mutations in the *ADSL* gene. These mutations partly decrease ADSL activity by affecting catalytic activity or stability [4,5]. In ADSL-deficient patients, dephosphorylated SAICAR and S-AMP accumulate in urine and cerebrospinal fluid, suggesting accumulation of SAICAR and S-AMP in cells. This molecular phenotype of ADSL deficiency is accompanied by epilepsy, seizures, delayed brain development, abnormal brain metabolism, and autism-like behaviors [6]. The molecular mechanism connecting ADSL activity and these phenotypes is still not fully understood, and in vivo models of this disease are being developed [7,8,9]. Nonetheless, ADSL represents a rare case where a single gene mutation is associated with an autism spectrum disorder [10].

In addition, ADSL activity is also connected to cancer in a complex manner. As a part of the de novo purine nucleotide biosynthesis pathway, partial loss of ADSL activity is expected to suppress the proliferation of cancer cells. Indeed, according to The Cancer Gene Atlas, the overexpression of ADSL is associated with unfavorable prognosis of liver cancer patients [11,12,13]. In some cells, genetic knockdown of ADSL expression interferes with cell proliferation [11,12,13]. For these types of cancer cells, inhibitors of ADSL might be beneficial. On the other hand, in colorectal cancer, overexpression of ADSL is associated with favorable prognosis. It may be because ADSL cleaves SAICAR, whose accumulation promotes growth factor-independent proliferation of cells [14,15,16]. Thus, ADSL activity can promote or suppress proliferation of cancer cells in a context-dependent manner.

Currently, there are several compounds that inhibit ADSL in vitro [17,18,19]. However, an ADSL inhibitor suitable for cellular and animal studies is not yet available. Because ADSL substrates and products are polar charged compounds, the active site of ADSL is also enriched with polar residues. For this reason, developing a cell-permeable inhibitor of ADSL is challenging.

Here, we report that NF-449 and its fragment 2,2’-(1,3-phenylenebis(carbonylimino))bisbenzenesulfonate (compound **1**, hereafter), inhibit ADSL in vitro. Compound **1** inhibits ADSL via a competitive mechanism. Compound **1** itself still bears a couple of sulfonates that limit cell permeability. Using the triethylammonium adduct of compound **1**, it was possible to inhibit ADSL in HeLa cells, probably because the positively charged triethylammonium shields the negatively charged sulfonate groups. With further improvement in its permeability, compounds derived from **1** might be useful for the study of ADSL in vivo and for suppressing the growth of ADSL-dependent cancer cells.

## 2. Materials and Methods

### 2.1. Small Molecules Compounds and Proteins

LOPAC^®^ 1280 is from Sigma-Aldrich (St. Louis, MO, USA; catalog number LO4200). NF449 octasodium salt was purchased from Torcis (Bristol, UK; catalog number 1391) and Santa Cruz Biotechnology (Dallas, TX, USA; sc-203159). SAICAR was from Toronto Research Chemicals (Toronto, ON, Canada; catalog number S688790) and MedChemExpress (Monmouth Junction, NJ, USA; catalog number HY-126585). S-AMP was from Sigma-Aldrich and Cayman Chemicals (Ann Arbor, MI, USA; catalog number 29696). Diphenylisophthalamide was from ChemBridge (San Diego, CA, USA; catalog number 5128450). All other chemicals were purchased from Sigma-Aldrich unless specifically noted otherwise. Plasmids encoding recombinant human ADSL were from Professor Roberta F. Colman (U. Delaware) and the Structural Genomics Consortium [20]. The protein was expressed and purified as reported by the Colman laboratory [21]. Because ADSL is not stable at 4 °C, all purification steps were carried out at ambient temperature as described in the literature [21]. Aliquots of purified protein solution were frozen in liquid nitrogen and stored at −80 °C for up to two months. Alternatively, the protein solution was kept at 37 °C in a capped tube for up to two weeks.

### 2.2. Screening of LOPAC^®^ 1280 Compounds

In each well of UV-transparent 384-well plates, a 45 μL solution containing 0.20 μM recombinant human ADSL and 2 μM screening compounds from the LOPAC 1280 library was placed. The reaction buffer contained 20 mM HEPES, pH 7.9, 50 mM KCl, 1 mM MgCl_2_, 1 mM DTT, and 1% (*v*/*v*) DMSO. Following a 30 min-long incubation at ambient temperature, the reaction was initiated by the addition of S-AMP (final: 0.20 mM). The reaction was monitored by measuring A_280_ every 10 min for 4 h at 37 °C.

### 2.3. ADSL Enzyme Activity Assay

ADSL activity assays were performed as described in the literature [21]. In brief, 0.60 mL solution containing recombinant human ADSL (0–2 μM), the test compound, 20 mM HEPES, pH 7.9, 50 mM KCl, 1 mM MgCl2, 1 mM DTT, and 1% DMSO was placed in a semi-micro quartz cuvette. This solution was kept at 37 °C for 30 min. The reaction was initiated by the addition of S-AMP or SAICAR to a final concentration of 0–2 mM (typically 0.20 mM). A280 was monitored every 5 s for 1 h using a Beckman DU650 spectrophotometer.

### 2.4. Synthesis of 2,2’-(1,3-Phenylenebis(carbonylimino))bisbenzenesulfonate (Compound ***1***)

To a solution of isophthaloyl chloride (Sigma-Aldrich I19403, 2.094 g, mol. wt. 203.02, 10.3 mmole) in 50 mL dichloromethane at 4 °C, 2-anilinesulfonic acid (Sigma-Aldrich A86805, 4.583 g, mol. wt. 173.19, 26.5 mmole) and triethylamine (Sigma-Aldrich T0886, 6 mL, mol. wt. 101.19, 43 mmole) were added. This solution was stirred at ambient temperature for 30 min, and volatile materials were evaporated by rotary evaporation. Residual materials were washed with deionized water, and the remaining materials were dissolved in methanol. Insoluble materials, mostly unreacted 2-anilinesulfonic acid, were removed by centrifugation at 1000 g for 5 min at 4 °C. The solution was dried under vacuum, yielding 5.34 g of the product as a dark brown-colored triethylammonium salt. For reverse-phase HPLC analysis, the product was dissolved in methanol. A 50 μL portion of this solution was injected into a Phenomenex Luna C18(2) (150 × 4.5 mm) connected to a Waters 1525 HPLC (solvent A: water with 0.1% formic acid, solvent B: acetonitrile with 0.1% formic acid; 0–5 min: 95%A, 5–30 min: 5–100% B, 30–35 min: 100% B, 35–40 min: 0–95%A, 40–45 min: 95%A, flow rate: 1 mL/min at RT, detection at 260 nm). For mass spectrometric analysis, the product dissolved in methanol was analyzed by ESI-qTOF (negative ion, acetonitrile with 10 mM triethylammonium bicarbonate as the eluent, flow injection analysis, Agilent G6546A) and *m*/*z* 475.0272 (calculated *m*/*z* for [M-H]^−^ 475.0270, error 0.4 ppm) was observed, along with peaks corresponding to the triethylammonium salt (calc. 576.1474, observed 576.1476) and the M-2H (calc. 237.0096, observed 237.0098) ions. NMR spectra were acquired using a Brucker 600 AV4 Neo instrument at the University of Kentucky Pharmacy NMR Center. The characterization data are available in the Supplementary Information accompanying the online version of this paper. The sodium salt of **1** was obtained by passing the solution of the triethylammonium salt through a cation exchange resin.

### 2.5. Blind Docking

The binding of NF-449 or compound **1** (in its anionic form) to the ADSL homotetramer (PDB: 2J91) was simulated using the Achilles Blind-Docking Server (https://bio-hpc.ucam.edu/achilles/ accessed on 20 October 2025) and Autodock Vina [22]. For this purpose, non-protein molecules in the PDB coordinate were removed before simulation. For the simulation with the Autodock Vina, the simulation was repeated five times using random seeds. Results using the Achilles server and the Autodock Vina were comparable to each other.

### 2.6. HeLa Cell Experiments

Cellular experiments and the measurement of nucleotides were carried out as we previously reported [15,16]. To measure the uptake of **1**, HeLa cells were grown in 10 cm dishes until they were approximately 50% confluent. Cells were incubated in Eagle’s Minimum Essential Medium supplemented with 10% fetal bovine serum, compound **1** (0–50 μM), and 0.1% DMSO for 1 h at 37 °C. The media were removed, and cells were washed five times with Dulbecco’s phosphate-buffered saline. Cells were lysed in ice-cold extraction solvent (95% methanol with 10 mM triethylammonium bicarbonate). The lysates were transferred to a centrifuge tube. Debris was removed by centrifugation at 150,000× *g* for 10 min at 4 °C. The supernatant was passed through Amicon Ultracel centrifugal filters (molecular weight cut-off 3 kDa). The filtrates were dried in vacuum and dissolved in 0.10 mL of extraction solvent. A 10 μL portion of this solution was subjected to HILIC-MS as we previously described [15,16]. To measure cell proliferation, cells were detached by trypsin-EDTA and counted using a Moxi-Z cell counter as we previously described [15,16].

## 3. Results

### 3.1. Identification of NF-449 as an ADSL Inhibitor

**Figure 2:**
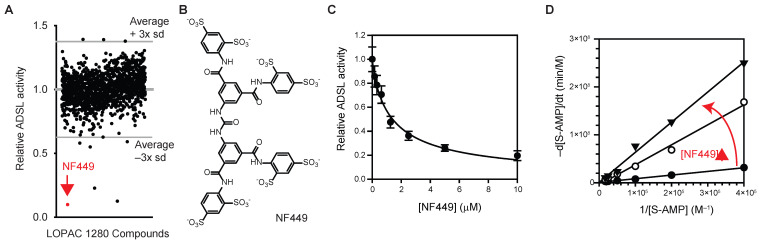
Identification of NF449 as an ADSL inhibitor. (**A**) Screening of LOPAC 1280 library compounds for ADSL inhibitors. (**B**) NF449. (**C**) Effect of NF449 on the activity of recombinant human ADSL measured using S-AMP as a substrate (avg. ± s.d., *n* = 3). (**D**) A Lineweaver–Burk plot showing that NF449 is a competitive inhibitor of ADSL.

To identify ADSL inhibitors, the effects of LOPAC^®^ 1280 library compounds on the activity of recombinant human ADSL were measured (Figure 2A). For this purpose, recombinant ADSL (0.2 µM) was incubated with LOPAC^®^ 1280 compounds (2 µM each) for 30 min at 37 °C, followed by the addition of S-AMP (final: 0.2 mM). ADSL activity was monitored by measuring changes in A_280_ for 4 h at 37 °C. Compounds inhibiting ADSL activity by more than three standard deviations (Z-score < −3, Figure 2A) were counted as hits. The result showed that NF-449 (Figure 2B), an antagonist of P2X1 receptors [23], might be an inhibitor of ADSL. NF-449 inhibited ADSL in a dose-dependent manner with an IC_50_ of 1.4 μM when either S-AMP or SAICAR was used (Figure 2C). NF-449 inhibited ADSL by a competitive mechanism (*K*_i_ 0.24 μM, Figure 2D).

In addition to NF449, two other compounds (amiodarone and *N*^ω^-hydroxyl-L-arginine) were also identified as potential ADSL inhibitors from this screen (Figure 2A). In subsequent tests, commercial amiodarone did not inhibit ADSL. ADSL became aggregated upon the addition of *N*^ω^-hydroxyl-L-arginine. These two compounds were not further studied.

### 3.2. Identification of an NF-449 Fragment as an ADSL Inhibitor

**Figure 3:**
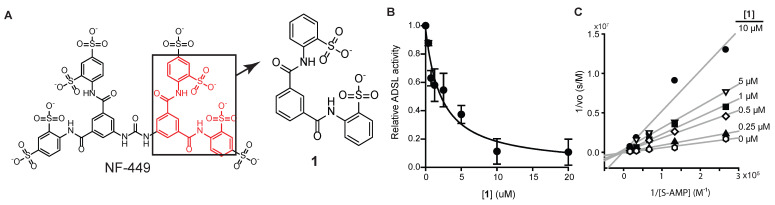
Inhibition of ADSL by a fragment of NF-449. (**A**) Structure of compound **1** in comparison to NF-449. (**B**) Inhibition of ADSL by 1 (avg. ± s.d., *n* = 3) measured using 0.2 mM S-AMP as a substrate. (**C**) A Lineweaver–Burke plot measured using S-AMP (0.2 mM) as a substrate.

**Figure 4:**
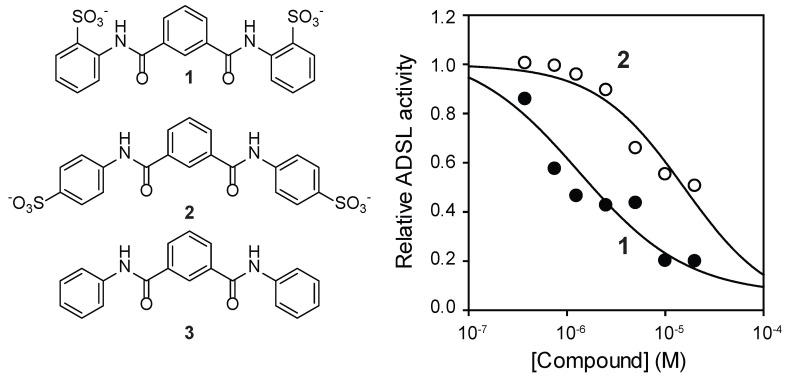
Effects of compounds related to **1** on ADSL.

NF-449 is a large (molecular weight >1320 g/mol) and highly polar (tPSA 615) compound. It is designed to regulate cell surface receptors and is unlikely to pass through cellular membrane. As NF-449 is unlikely to inhibit intracellular targets because of its size and charge, we thought that a smaller fragment of NF-449 might be used to inhibit ADSL in cells. For this purpose, compound **1** (molecular weight 474, tPSA 173, logS-5.8; Figure 3A and Supplementary Information Figures S1–S5) and other fragments of NF-449 were synthesized. Compound **1** inhibited human ADSL in a dose-dependent manner with an IC_50_ value of 2 μM (Figure 3B). Like NF-449, **1** was a competitive inhibitor of ADSL (Figure 3C). The *K*_i_ was only marginally higher than that of NF449 (*K*_i_ 0.4 μM). Compound **1** also inhibited ADSL when SAICAR was used as a substrate. 4,4’-(Isophthaloylbis(azanediyl))dibenzenesulfonate (compound **2**) also inhibited ADSL but with a higher *K*_i_ (15 μM) than **1** (Figure 4). Commercially available *N, N’*-diphenylisopthalamide (compound **3**), which lacks sulfonate groups of **1**, did not inhibit ADSL at the concentrations tested (up to 30 μM).

**Figure 5:**
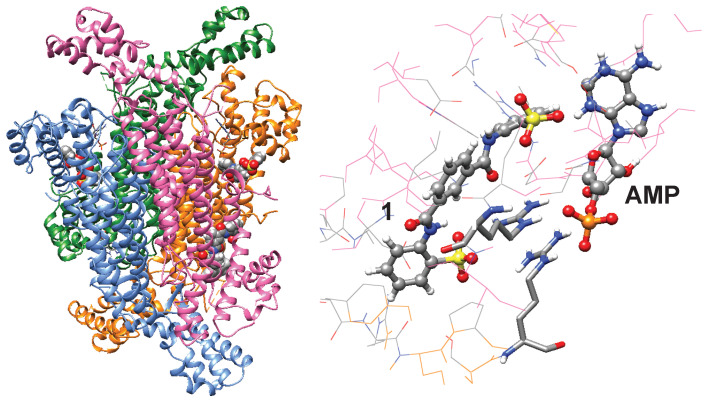
Potential mode of interaction. A blind-docking simulation of compound **1** to a tetrameric structure of ADSL [25] is shown. Active sites are noted with AMP.

To better understand how compound **1** might inhibit ADSL, a blind-docking simulation [24] was performed (Figure 5). Simulations were carried out using the Achilles Blind-Docking Server (https://bio-hpc.ucam.edu/achilles/ accessed on 20 October 2025). The top four predicted poses showed that compound **1** bind to pockets adjacent to the active sites. A sulfonate group of **1** is predicted to interact with two arginine residues that interact with the phosphate groups of substrates and products. This model may explain how **1** competitively inhibits ADSL. Simulation of NF-449 suggested that NF-449 might bind to the active site cleft (Figure S6).

### 3.3. Cellular Effects of Compound ***1***

**Figure 6:**
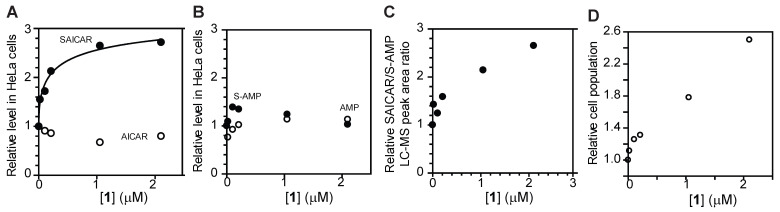
Compound **1** inhibits ADSL in HeLa cells. (**A**,**B**) Relative levels of SAICAR, AICAR, S-AMP, and AMP in HeLa cells treated with varying concentrations of 1 for 12 h. (**C**) The ratio of SAICAR to S-AMP in HeLa cells treated with 1 for 12 h. (**D**) Proliferation of HeLa cells in medium supplemented with dialyzed medium and varying concentrations of 1 for two days.

Next, we asked whether **1** inhibits ADSL in mammalian cells. For this purpose, HeLa human cervical carcinoma cells were incubated with varying concentrations of compound **1**, in its triethylammonium salt form, for 12 h. Cells were lysed, and the amounts of SAICAR and S-AMP were measured by HILIC-HPLC-MS as we previously reported [15,16] (Figure 6). Compound **1** increased the cellular level of SAICAR by approximately 2.5-fold with an EC_50_ of 0.4 µM (Figure 6A–C). The level of AICAR was approximately 30% lower, which is consistent with partial inhibition of ADSL in cells. Compound **1** had less effect on the cellular level of S-AMP and AMP (Figure 6B). Compound **1** also increased the ratio of SAICAR to S-AMP, which is commonly used to assess the severity of ADSL deficiency (Figure 6C). Compound **1** also promoted the mitogen-independent proliferation of HeLa cells (Figure 6D). Taken together, these results indicate that **1** is permeable to cultured cells and suitable for the study of ADSL in cells. The compound, however, has limited permeability through blood–brain barrier in vitro and might not be suitable for the study of ADSL in animal brains (Figure S7).

## 4. Discussion

In this paper, we report the generation of a cell-permeable ADSL inhibitor. From a high-throughput screening, NF-449 was identified as an ADSL inhibitor. Because ADSL itself is unlikely to inhibit intracellular ADSL, smaller fragments of NF-449 were tested. Compound **1**, a smaller fragment of NF-449, inhibited purified recombinant ADSL with a *K*_i_ similar to the one shown by NF-449. Because **1** has fewer charges than NF-449, it was expected to be better permeable through cellular membranes. Indeed, the triethylammonium salt of **1** was efficiently transported into cells and inhibited cellular ADSL. This compound thus represents a novel class of a cell-permeable ADSL inhibitor. It might be used to study the role of ADSL in cultured cellular models of ADSL deficiency. However, the permeability of **1** through the blood–brain barrier appears to be limited. The permeability of **1** may be further improved, possibly by replacing the sulfonate groups, making a reagent useful for the study of ADSL in the brain possible.

## Data Availability

The original contributions presented in this study are included in the article. Further inquiries can be directed to the corresponding author.

## Supplementary Materials

The following supporting information can be downloaded at: https://www.mdpi.com/article/10.3390/mps8060126/s1, Figure S1: Reverse-phase HPLC chromatograph of **1**; Figure S2: ESI-TOF mass spectrum of **1**; Figure S3: ^1^H-NMR spectrum of **1**; Figure S4: ^13^C-NMR spectrum of **1**; Figure S5: Decoupled HSQC spectrum of **1**; Figure S6: A simulated structure of ADSL tetramer (PDB: 2J91) bound to NF-449; Figure S7: Permeability of **1** through an in vitro model of blood–brain barrier.

## Abbreviations

The following abbreviations are used in this manuscript:

ADSL	Adenylosuccinate lyase
EC_50_	Half-maximal effective concentration
SAICAR	N-Succinyl-5-aminoimidazole-4-carboxamide-1-ribose-5’-monophosphate
AICAR	5-Aminoimidazole-4-carboxamide-1-ribose-5’-monophosphate
S-AMP	Adenylosuccinate
AMP	Adenosine 5’-monophosphate
