# Supplementary material for: Development of Cell-Permeable Adenylosuccinate Lyase Inhibitor
Source: Methods Protoc. 2025 Oct 29;8(6):126. doi: 10.3390/mps8060126 (PMC12641998; doi:10.3390/mps8060126)
Supplement: Supplementary file 1 [file mps-08-00126-s001.zip › mps-3872467-supplementary.pdf]

Supplementary Information

Development of cell-permeable adenylosuccinate lyase inhibitor

Yijia Hu [1], and Young-Sam Lee [1,2,*]

[1] Department of Biology, Johns Hopkins University, Baltimore, Maryland 21218, United States.
[2] Department of Molecular and Cellular Biochemistry, University of Kentucky, Lexington, Kentucky 40536, United States.
* Correspondence: young-sam.lee@uky.edu

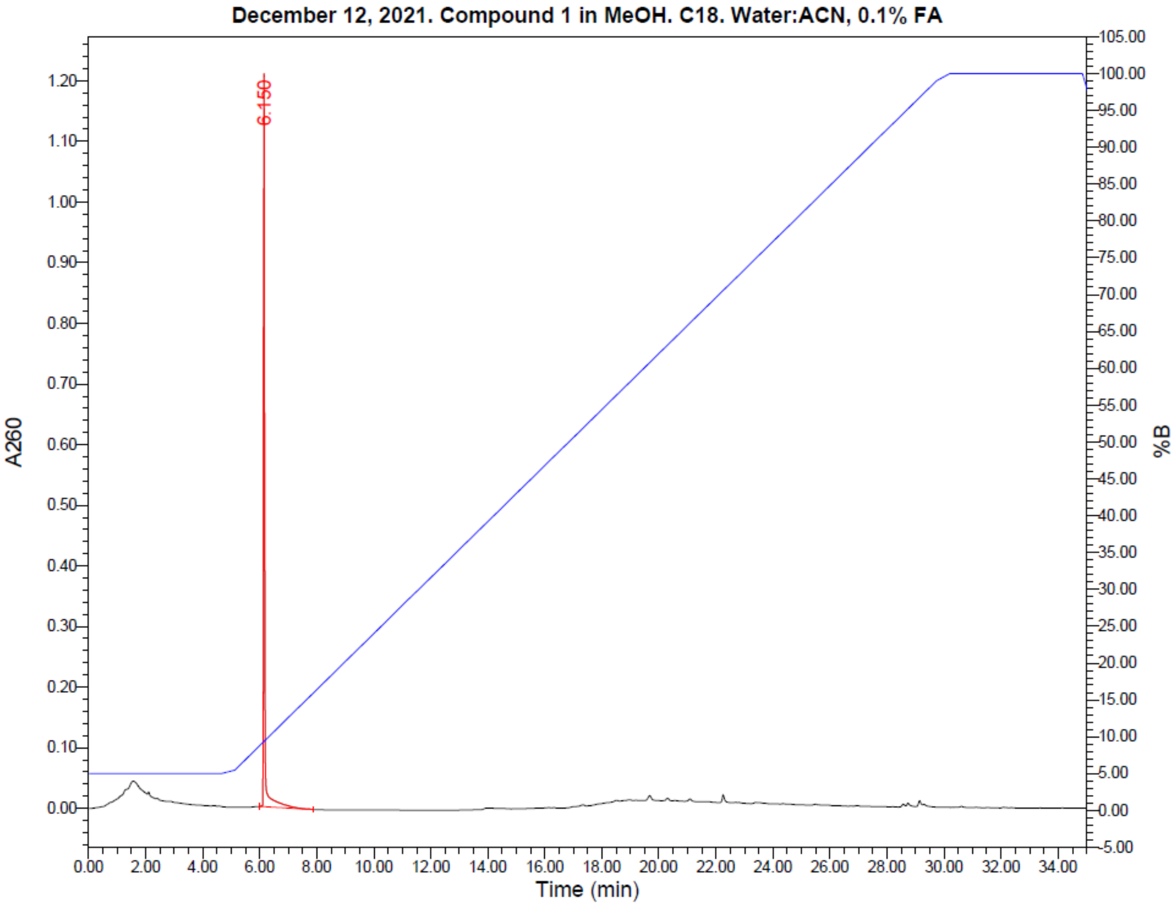

Figure S1. Reverse phase HPLC chromatograph of compound **1**. The compound in methanol was injected to a C18 reverse phase column and eluted with a gradient (blue line) of water and acetonitrile (0.1% vol./vol. formic acid). The compound, based on the peak area at 260 nm, was eluted at 6.15 min and at least 95% pure.

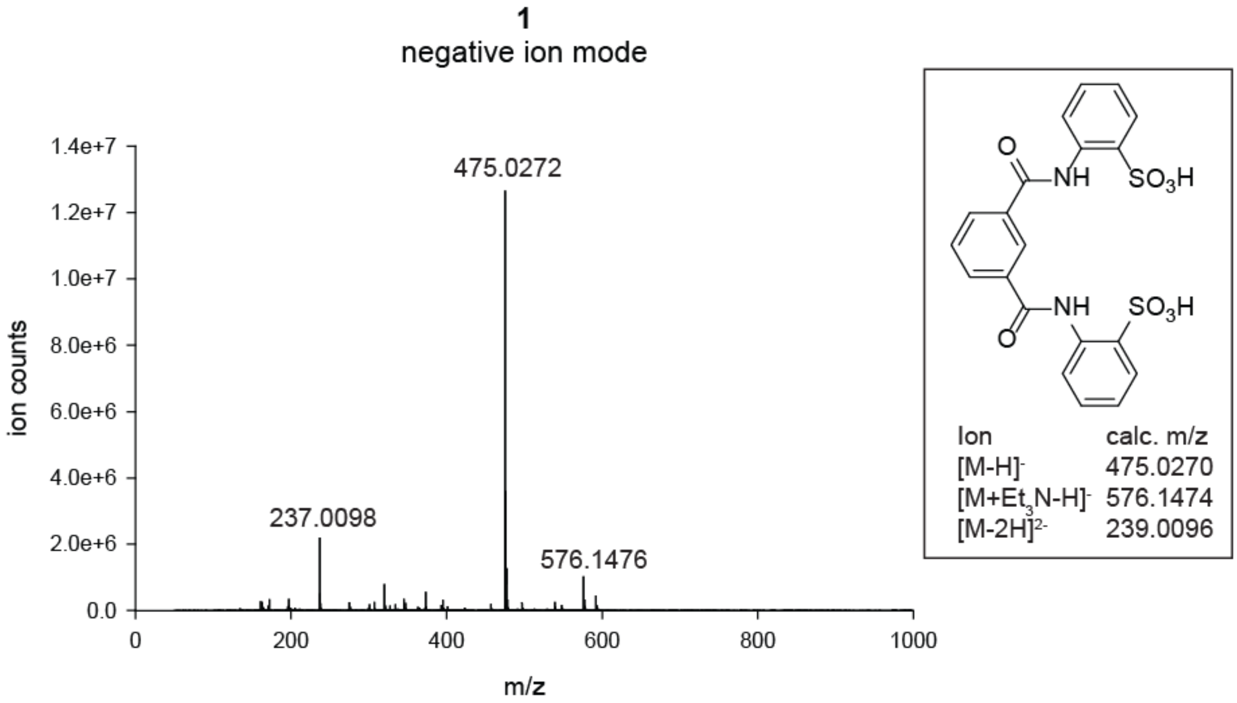

Figure S2. ESI-TOF mass spectrum of **1** (negation ion mode, ESI-TOF, flow injection analysis). The calculated m/z (M-H ion) is 475.0270, while the observed m/z was 475.0272.

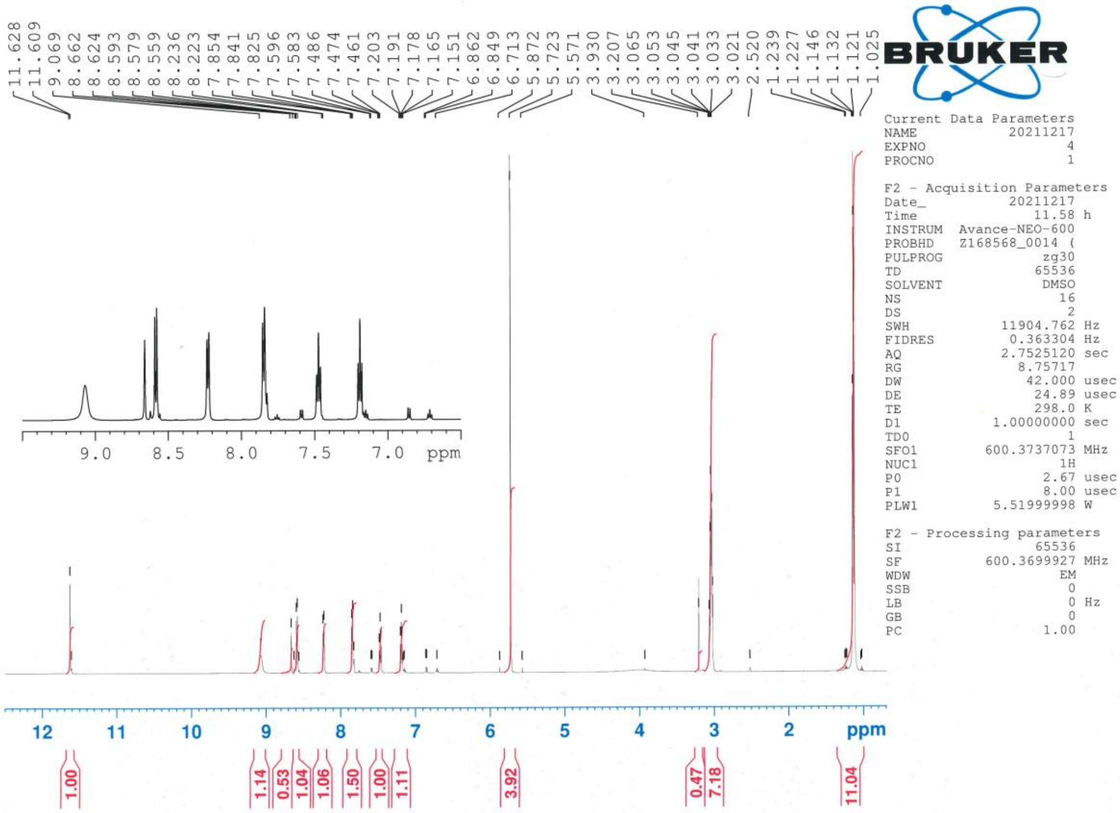

Figure S3. ^{1}H-NMR spectrum of 1 (DMSO-d_6, 600 MHz)

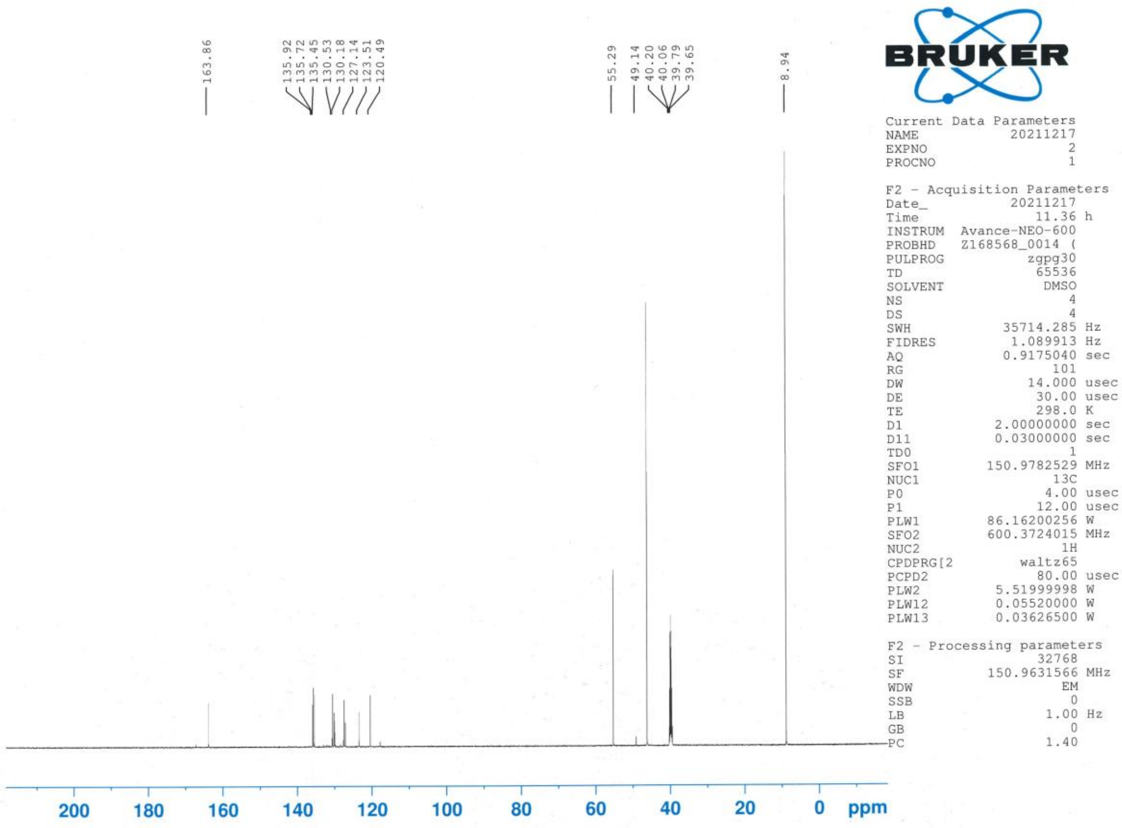

Figure S4. ^{13}C-NMR spectrum of **1** (DMSO-d_6).

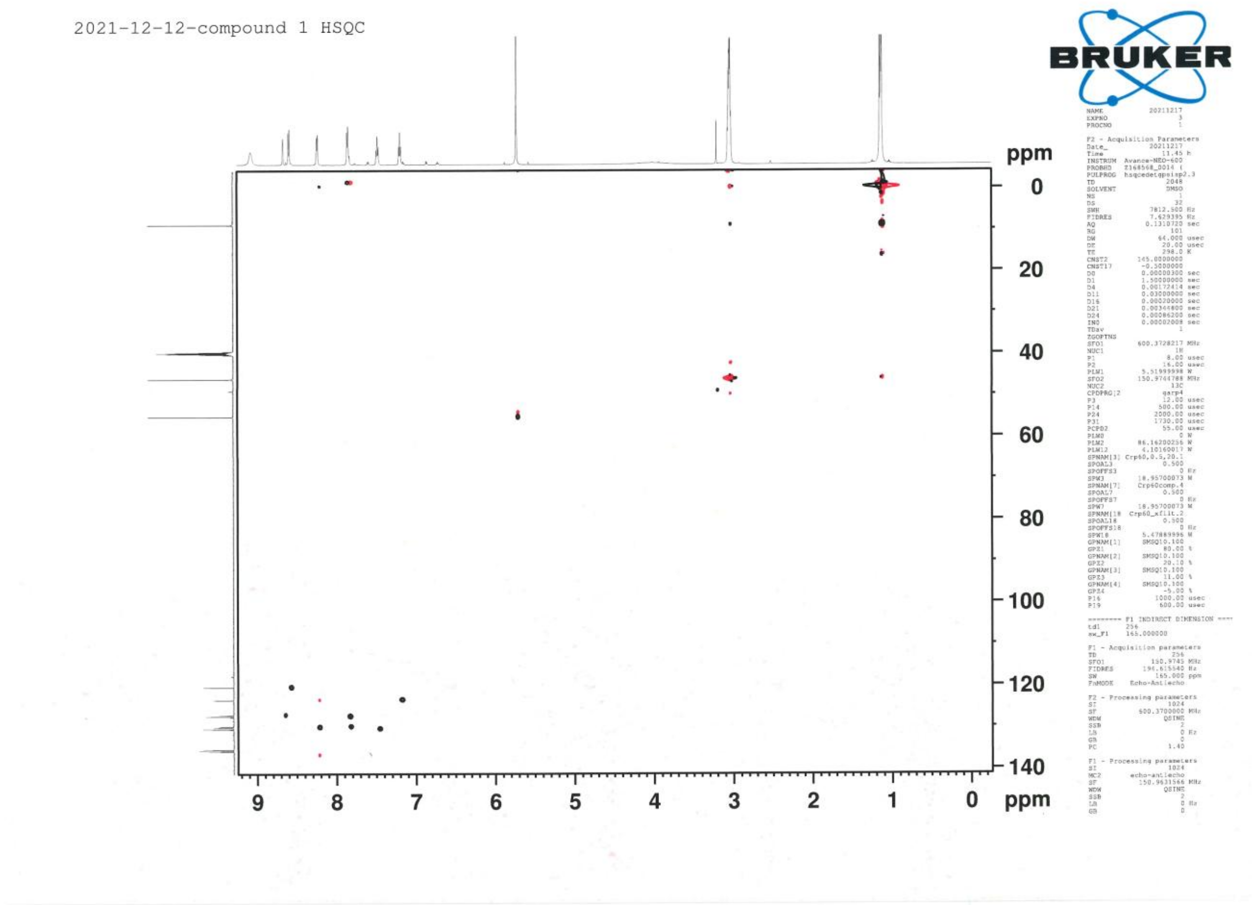

Figure S5. Decoupled HSQC spectrum of **1**.

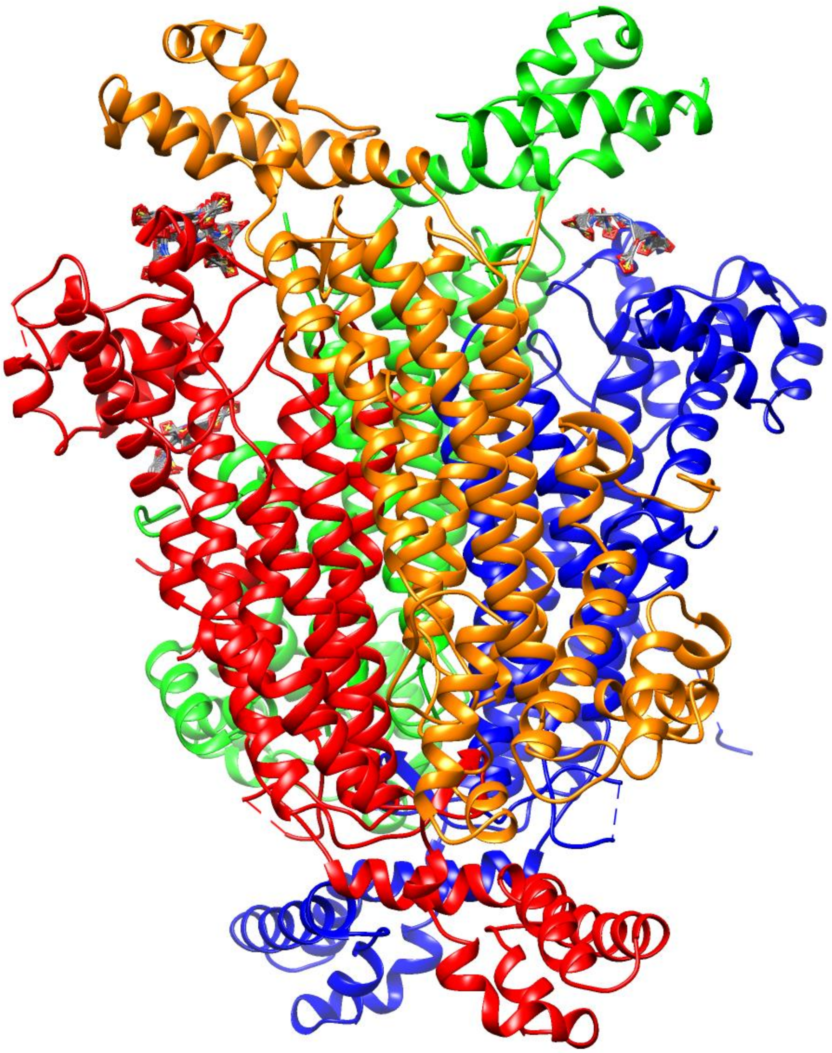

Figure S6. A simulated structure of ADSL tetramer (PDB: 2J91) bound to NF-449 (protein chains are shown in ribbons, and top 10 predicted modes of NF-449 are shown as sticks).

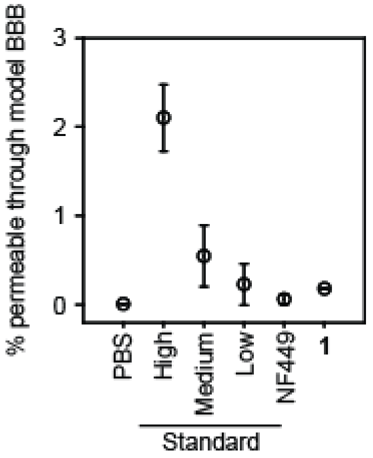

Figure S7. Permeability of compounds through an *in vitro* model of blood-brain-barrier (BBB) (avg. ± s.d., n =3). Compound **1** has a BBB-permeability comparable to the low-permeability standard.
